# Predictors associated with inappropriate transport of near shore spinal injuries

**DOI:** 10.1016/j.cjtee.2021.05.001

**Published:** 2021-05-15

**Authors:** Tucker Lurie, Timothy Traynor, Maira Sher Bano, Quincy K. Tran

**Affiliations:** a University of Maryland School of Medicine, Baltimore, MD, 21201, USA; b Research Associate Program in Emergency Medicine & Critical Care, University of Maryland School of Medicine, Baltimore, MD, 21201, USA; c Department of Emergency Medicine, University of Maryland School of Medicine, Baltimore, MD, 21201, USA; d Program in Trauma, The R Adams Cowley Shock Trauma Center, University of Maryland School of Medicine, Baltimore, MD, 21201, USA

**Keywords:** Transport, Spinal injury, Near shore spinal injury, Wave riding, Shallow water diving

## Abstract

**Purpose:**

Spinal injuries resulting in neurological damage cause significant morbidity. Swift neurosurgical intervention can mitigate negative outcomes. However, variable mechanisms of injury may be associated with inappropriate transport (IAT), which may delay necessary surgical interventions. Patients with near shore spinal injuries (NSSI) presented with unique mechanisms, so we investigated factors associated with IAT in patients with NSSI.

**Methods:**

We performed a multicenter retrospective study of all adult patients transported from a beach resort to 3 hospitals for suspected NSSI between 2006 - 2017. We excluded patients transferred to other facilities, and those not injured in the water. Primary outcome was IAT, defined as patients with NSSI requiring transfer to another trauma center. To avoid heterogeneity in our analysis, we further excluded patients without NSSI who were inappropriately transported to a level I trauma center. We used multivariable logistic regression to assess association of independent variables (such as demographic, environmental, and clinical factors) with outcome.

**Results:**

We analyzed 278 patients with suspected NSSI, and found 14 (5.0%) had IAT. Compared to appropriately transported patients, diving was associated with higher percentages of IAT (28.6% *vs.* 3.9%, *p* = 0.014) and more were transported by air (50.0% *vs.* 20.6%, *p* = 0.01). In multivariable regression, patients’ oxygenation saturation (odds ratio [OR] = 0.8, 95% confidence intervals [*CI*]: 077–0.98) and diving (*OR* = 7.5, 95% *CI*: 1.2–46) were significantly associated with IAT.

**Conclusion:**

Rate of IAT for patients with NSSI was low. However, first responders and emergency medicine providers should be aware that diving is associated with a higher likelihood of IAT.

## Introduction

Beaches are popular tourist sites for recreational activities. A recent study suggested that overall beach injuries range from 1.3% to 12% of beachgoers.^1^ Traumatic spinal injury, one of the most feared beach injuries, is significantly associated with burden when resulting in neurologic damage.^2^

A previous study showed early surgical intervention for spinal cord injury was associated with improved recovery of neurological deficits.^3^ Unfortunately, patients who required interhospital transfer were more likely associated with delayed surgical interventions. Therefore, early detecting and appropriately transporting patients to a facility with neurosurgical capability is crucial in determining outcomes. However, there is a balance for first responders and transport teams to decide where to transport patients. Bringing patients without injuries to a faraway trauma center, especially by air, may increase cost to patients and society.^4^ On the other hand, transporting patients with significant injuries to local non-trauma emergency departments (EDs) may delay necessary interventions.

The current Maryland Institute for Emergency Medical Services Systems transport protocols dictate that any patient with paraplegia or quadriplegia be taken to a local trauma center.^5^ Additional trauma transports include patients with high-energy/high-risk mechanism, or elderly patients (>55 years) with low-impact mechanism. Maryland Institute for Emergency Medical Services Systems Guidelines do not directly address transport protocols such as air or ground transport. No transport protocols for signs and symptoms regarding near shore spinal injury (NSSI) exist. Because NSSI involves a relatively low-speed mechanism, it is likely that inappropriate transport (IAT) is more frequent when compared to high-speed mechanisms like motor vehicle collisions*.*

Our study aimed to investigate the incidence and factors associated with IAT in patients sustaining NSSI at one of our state's popular beach resorts. We evaluated the transport patterns of patients from the beach in Ocean City, Maryland to nearby hospitals for suspected spinal injuries. The Ocean City Beach Patrol (OCBP) guards the 9-mile shoreline every day from May to September and collects data regarding thousands of injury reports during the season. Most trauma patients from Ocean City's beach are transported to one of three hospitals: Atlantic General Hospital (AGH), a non-trauma center 13 km from the beach, Peninsula Regional Medical Center (PRMC), a Trauma Level II 50 km from the beach, and University of Maryland Shock Trauma Center (STC), a regional Level I trauma center 220 km from the beach.

## Methods

### Study settings

This is a multicenter retrospective study of all adult patients transported from the beach in Ocean city, Maryland to one of three hospitals for evaluation of suspected NSSI. Patients transported to one of the study hospitals from the beach for evaluation of suspected NSSI between 2006 and 2017 were eligible. Our study was approved by the Institutional Review Board at STC, PRMC and the Ethics Committee at AGH.

We first obtained data from the OCBP's database of all incidents from 2006 to 2017. From the database, we identified patients' demographic data, mode of transport from the beach, date and time of transport, and name of the destination hospitals. We subsequently followed these patients to the corresponding transport location and matched them with the records at the destination hospitals.

Clinical data were collected from the initial receiving hospitals’ ED records, including vital signs, history of present illness, past history of spinal disease (e.g., spinal stenosis, osteophytes), and results from physical examinations and imaging studies. Spinal injury was defined as any damage to the spinal bones, ligaments, or cord as detected on CT or MRI. Spinal cord injury was defined as any contusion, hematoma, or transection of the cord. Muscle strains were not considered spinal injury for this study.

### Patient selection

Adult patients were eligible if the OCBP incidence reports described any neck/back stabilization (c-collar, backboard), any complaints of neck or back pain or numbness/tingling of extremities. Patients were excluded if they had cardiac arrests because we were interested in identifying patients’ chief complaints and physical findings as predictors of IAT. Additionally, patients were excluded if records were missing or they were transported to hospitals other than AGH, PRMC, or STC.

### Data collection

The investigators travelled to the sites to collect data from each hospital. One investigator, who was not blinded to the study hypothesis, was trained by the principal investigator for data collection. Data were collected using a standardized Microsoft Access database (Microsoft Corporation, WA). Data regarding spinal injuries were double-checked by the principal investigator. Discrepancies were resolved by agreement among the investigators.

### Outcome

Primary outcome was the incidence of IAT of patients with spinal injuries. IAT was defined as a patient with spinal injury who required transfer to a second hospital for higher level of management. Patients without spinal injury who were initially transported to a higher-level center represented another form of IAT, but we excluded these patients in the analysis due to concerns of heterogeneity among these two populations.

### Statistical analysis

We did not perform sample size calculation as the true incidence of IAT in this unique patient population is unknown. We remedied the situation by trying to include all possible patients from multiple hospitals and as far back as records were available.

We first used descriptive data to compare characteristics of patients with appropriate and IAT. Continuous data were expressed as mean and SD or median and interquartile range (IQR) as appropriate. Student *t*-test or Mann Whitney *U* test was used to analyze groups’ means or median as appropriate. We used Chi-square with Yates correction to analyze categorical data.

We performed a multivariable logistic regression to assess the association between patients' demographic and clinical factors and our primary outcome. To identify relevant independent variables for the multivariable logistic regression, we first performed univariate logistic regression for each independent factor and the outcome. In addition to a priori-determined clinically significant factors, we added those independent variables with *p* value ≤ 0.10 into our multivariable logistic regression. The goodness-of-fit of our multivariable logistic regression model was assessed by the Hosmer-Lemeshow test. Modelling with Hosmer-Lemeshow test's *p* value > 0.05 was considered good fit. All data were analyzed by Minitab version 18 (State College, PA, USA). Data with two-sided *p* value < 0.05 were considered statistically significant.

## Results

### Patients’ demographic characteristics

**Fig. 1:**
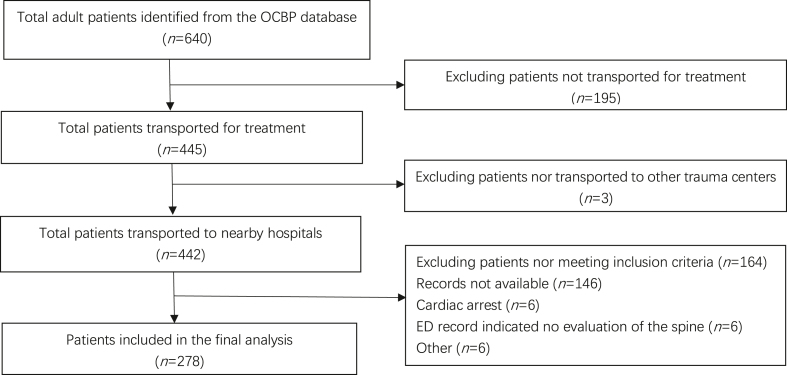
Patient selection diagram. OCBP: Ocean City Beach Patrol; ED: emergency department.

Over the 12-year period, the OCBP database identified 640 suspected spinal injuries in adult patients. Among 445 patients being transported to hospitals for evaluation, 442 were transported to the 3 study sites. We included 278 patients with sufficient medical records for analysis. We excluded 162 patients, of whom 156 did not have sufficient records, and 6 were unable to report symptoms. Fig. 1 shows the patient selection diagram.

There were 14 (5.0%) patients who had spinal injury and were inappropriately transported. Characteristics of patients with appropriate transport *vs.* IAT were similar (Table 1). Mean age (SD) for inappropriately transported patients was 46 (18) years compared with 42 (14) years for appropriately transported patients (*p* = 0.30). Percentage of male patients was similar between inappropriately transported group (92.9%) *vs.* appropriately transported group (76.3%) (*p* = 0.20). There was higher percentage of patients who sustained injuries after diving in the inappropriately transported group (28.6%) *vs.* the appropriately transported group (3.9%) (*p* < 0.001).

**Table 1:** Comparison of demographic and environmental factors between patients who were inappropriately *vs.* appropriately transported for evaluation of suspected near shore spinal injuries.

Variables	IAT (*n* = 14)	Appropriate transport (*n* = 257)	*p* value
Age (years), mean (SD)	46 (18)	42 (14)	0.31
Gender, *n* (%)			0.20
Male	13 (92.9)	196 (76.3)	
Female	1 (7.1)	61 (23.7)	
Race, *n* (%)			0.23
White	10 (71.4)	222 (86.4)	
Non-white	4 (28.6)	35 (13.6)	
Distance from home zip to first hospital (km), mean (SD)	230 (132)	266 (338)	0.69
Home zip median income, (in 1000s), mean (SD)	71.4 (24)	78.4 (23)	0.26
Transport during rush hour, *n* (%)	4 (28.6)	38 (14.8)	0.47
Transportation mode, *n* (%)			**0.02**
By air	7 (50.0)	53 (20.6)	
By ground	7 (50.0)	204 (79.4)	
First hospital of transport, *n* (%)			
Non-trauma ED	6 (42.9)	130 (50.6)	0.70
Level II trauma center	8 (57.1)	92 (35.8)	0.24
Regional level I trauma center	0	35 (13.6)	NA
Time to appropriate facility (min), median (IQR)	329 (299–381)	59 (46–70)	**<0.001**
Water activities, *n* (%)			
Body surfing	5 (35.7)	110 (42.8)	0.76
Boogie boarding	3 (21.4)	85 (33.1)	0.66
Shallow water diving	4 (28.6)	10 (3.9)	**<0.001**
Swimming	1 (7.1)	19 (7.4)	0.99
Other (rafting, standing)	1 (7.1)	33 (12.8)	0.86
Shore break waves, *n* (%)	11 (78.6)	206 (80.2)	0.95
Water temperature (°C), mean (SD)	22 (2)	22 (2)	0.99
Wave heights (meters), mean (SD)	1 (0.4)	0.9 (0.3)	0.23

Note: Shore break waves refers to waves that crash directly on the shore as opposed to over deep water.

Bolded *p* value denoted statistical significance.

IAT: inappropriate transport; NA: not available; ED: emergency department; IQR: interquartile range; SD: standard deviation.

### Patients’ clinical characteristics

Patients who were inappropriately transported had higher rates of any spinal injury (100.0% *vs.* 34.2%, *p* < 0.001) (Table 2) or any spinal injury at the cervical level (85.7% *vs.* 28.4%, *p* < 0.001).

**Table 2:** Comparison of clinical factors between patients who were inappropriately *vs.* appropriately transported for evaluation of suspected near shore spinal injuries.

Variables	IAT (*n* = 14)	Appropriate transport (*n* = 257)	*p* value
ESI, median (IQR)	3 (2–3)	3 (2–3)	0.99
Triage SBP, mean (SD)	142 (26)	139 (22)	0.62
Triage HR, mean (SD)	77 (19)	76 (14)	0.80
Triage pain, mean (SD)	6 (4)	5 (3)	0.23
Triage GCS, median (IQR)	14 (3)	15 (1)	**0.002**
Triage RR, mean (SD)	17 (5)	18 (3)	0.24
Triage SpO_2_, mean (SD)	96 (5)	98 (2)	**0.001**
Triage temperature (°C), mean (SD)	37 (1)	37 (0.5)	0.99
Shock index^a^, mean (SD)	0.6 (0.2)	0.6 (0.1)	0.99
Pre-existing spinal cord conditions, *n* (%)	6 (42.8)	110 (42.8)	0.99
Spinal injuries^c^, *n* (%)			
Any spinal injury^b^	14 (100.0)	88 (34.2)	**<0.001**
Cervical spine	12 (85.7)	73 (28.4)	**<0.001**
Thoracic spine	1 (7.1)	9 (3.5)	0.89
Lumbar spine	2 (14.3)	8 (3.1)	0.55
Any spinal cord injury^b^	1 (7.1)	40 (15.6)	0.81
Cervical spine	1 (7.1)	38 (14.8)	0.83
Thoracic spine	0 (0)	0 (0)	
Lumbar spine	0 (0)	2 (0.8)	
Chief ED complaints^b^, *n* (%)			
Pain	9 (64.3)	191 (74.3)	0.51
Numbness or tingling	4 (28.6)	74 (28.8)	0.99
Weakness or paralysis	4 (28.6)	33 (12.8)	0.40
Loss of consciousness	1 (7.1)	22 (8.6)	0.95
Location of symptoms^b^, *n* (%)			
Head (pain)	1 (7.1)	40 (15.6)	0.81
Back (pain)	0 (0)	48 (18.7)	
Extremities (pain/tingling)	6 (42.9)	85 (33.1)	0.62
Neck (pain)	7 (50.0)	101 (39.3)	0.57

Bolded *p* value denotes statistical significance.

IAT: inappropriate transport; ESI: emergency severity index; IQR: interquartile range; SBP: systolic blood pressure; HR: heart rate in beats per minutes; SD: standard deviation; GCS: Glasgow coma scale; RR: respiratory rate per minute; SpO_2_: pulse oximeter measuring oxygen saturation in percent; ED: emergency department.

Note:

a Shock index was calculated from lowest systolic blood pressure and concurrent pulse during ED stay.

b One patient may have more than one condition.

c Diagnosed at hospital via radiographic evidence.

The median time interval from injury to appropriate facility was significant longer for inappropriately transported patients at 329 min (IQR: 299–381), compared with 59 min (IQR: 46–70, *p* < 0.001) for appropriately transported patients. Inappropriately transported patients were also more likely to be transported by air (50.0% *vs.* 20.6%, *p* < 0.01).

### Variables associated with IAT

Our multivariable logistic regression (Table 3) revealed 2 factors associated with IAT. Patients who sustained injury after shallow water diving was associated with a significantly higher likelihood of IAT (odds ratio [*OR*] = 7.5, 95% confidence intervals [*CI*]: 1.2–46, *p* = 0.042). Patient who presented with higher oxygen saturation was associated with 80.0% less likelihood of IAT (*OR* = 0.8, 95% *CI*: 0.77–0.98, *p* = 0.047).

**Table 3:** Multivariable logistic regressions assessing association between clinical factors and inappropriate transport of patients with suspected nearshore spinal injuries.

Variables	Univariate analysis			Multivariable analysis^#^		
	*OR*	95% *CI*	*p* value	*OR*	95% *CI*	*p* value
Transport type - air	3.6	1.2–11	0.024	2.9	0.8–11	0.12
Presenting SpO_2_	0.78	0.6–0.91	0.006	0.80	0.77–0.98	**0.047**
ED complaint						
Numbness or tingling∗	0.97	0.3–3.2	0.96	0.71	0.17–3.1	0.64
Weakness or paralysis∗	2.6	0.8–8.8	0.15	0.63	0.4–11	0.75
Loss of consciousness∗	0.77	0.1–6.1	0.79	0.47	0.05–4.5	0.48
Any neuro deficit∗	1.9	0.5–7.2	0.37	0.63	0.03–11	0.75
Diving	10	2.7–38	0.003	7.5	1.2–46	**0.042**
Alcohol use	6.9	1.7–28	0.02	3.2	0.4–23	0.28
ED intubation	9.2	2.5–34	0.003	2.2	0.3–15	0.44

Note: ∗ Clinically significant factor.

^#^ Hosmer-Lemeshow Test Chi-square 8.68, *p* = 0.19.

Bolded factors denoted statistically significance. Only clinically significant factors and independent variables with *p* value ≤ 0.10 were included in the multivariable logistic regression.

*OR:* odds ratio; *CI:* confidence interval; SpO_2_: pulse oximeter measuring oxygen saturation in percent; ED: emergency department.

## Discussion

The rate of IAT in our patient population was small, with over 92% of patients appropriately transported to their first destination hospital. However, our study identified 2 factors associated with IAT of patients with spinal injuries: shallow-water diving and patient's presenting oxygen saturation.

Shallow water diving has been noted as a high-risk activity for spinal cord injuries, particularly in the cervical spine.^6^, ^7^, ^8^ Shallow-water diving has mostly been studied in pools^6^, ^7^, ^8^ but is also common at rivers and the ocean. Shallow-water diving injury typically results in hyperextension or hyperflexion, but direct contact with the top of the head can cause compression fractures as well. Overall, triage protocols have low sensitivity for severely injured patients,^9^ and our state emergency medical service (EMS) protocol did not list shallow water diving as a high-risk mechanism,^5^ on-scene responders may not recognize the severity of injury in these patients. Clinicians should be cognizant that patients who sustained injuries while involved in shallow-water diving are at high-risk for spinal injury. These patients should be considered for direct transport to a facility with neurosurgical capability.

Our analysis also showed that patients with high oxygen saturation were less likely to be inappropriately transported. We hypothesized that transport teams would prioritize transport of hypoxic patients to the nearest facility for stabilization and interventions before bringing them to a facility with neurosurgical capability. As a result, transport of these patients was clinically indicated and appropriate.

In addition to clinical factors, we aimed to study demographic and environmental factors that may be associated with IAT. Notably, socioeconomic status (median income of patients’ home zip was used as a proxy for socioeconomic status), local *vs.* non-local status, age, race, sex, and transport during rush hour were included in the analysis. None of these factors were associated with IAT in our multivariable regressions. The high rate of appropriate transport, regardless of other demographic influences, suggested that pertinent clinical information was the most important factor directing transport decisions.

Our study has several limitations. Although we included a large number of patients with suspected NISSs, the sample size of patients with true NISSs associated with IAT was still small. This small sample size likely affected our outcomes, such as the wide 95% *CI* for the independent variable of diving. Furthermore, a large percentage of EMS documentation was missing so we were not able to assess the immediate medical decision-making process from EMS personnel. We also did not have access to many patients' in-hospital records to assess patients' management and outcomes, although this information should not affect emergency providers and EMS personnel's decision to transport patients to any facilities.

Overall, the number of IAT of patients with NISSs was small. While many IATs may be acceptable, clinicians should be cognizant that shallow-water diving is associated with higher likelihood of injury and inappropriate triage and transport.

## Funding

This research was supported by an intramural grant from the Department of Emergency Medicine at the University of Maryland School of Medicine.

## Ethical statement

Our study was approved by the Institutional Review Board at STC, PRMC and the Ethics Committee at AGH.

## Declaration of competing interest

The authors declared no conflict of interest.

## References

[1] Doelp M.B., Puleo J.A., Cowan P. (2018). Delaware coast Delaware surf zone injury demographics. Am J Emerg Med.

[2] Hall O.T., McGrath R.P., Peterson M.D. (2019). The burden of traumatic spinal cord injury in the United States: disability-adjusted life years. Arch Phys Med Rehabil.

[3] Lee D.Y., Park Y.J., Song S.Y. (2018). The importance of early surgical decompression for acute traumatic spinal cord injury. Clin Orthop Surg.

[4] Kashefi N, Dissanaike S. Use of air transport for minor burns: is there room for improvement? J Burn Care Res. 37:e453-460. doi: 10.1097/BCR.0000000000000276. 10.1097/BCR.0000000000000276 26284627

[5] EMS Provider Protocol. Maryland Institute for Emergency Medical Services Systems. http://www.miemss.org/home/ems-providers/protocols. Web accessed 10/07/2019.

[6] Vlok A.J., Petersen J., Dunn R.N. (2010). Shallow-water spinal injuries--devastating but preventable. S Afr Med J.

[7] Chang S.K.Y., Tominaga G.T., Wong J.H. (2006). Risk factors for water sports-related cervical spine injuries. J Trauma.

[8] Aito S., d'Andrea M., Werhagen L. (2005). Spinal cord injuries due to diving accidents. Spinal Cord.

[9] Voskens F.J., van Rein E.A.J., van Der Sluijs R. (2018). Accuracy of prehospital triage in selecting severely injured trauma patients. JAMA Surg.

